# SDE5, a putative RNA export protein, participates in plant innate immunity through a flagellin-dependent signaling pathway in *Arabidopsis*

**DOI:** 10.1038/s41598-017-07918-x

**Published:** 2017-08-29

**Authors:** Mohammad Nazim Uddin, Salina Akhter, Rupak Chakraborty, Ji Hyeong Baek, Joon-Yung Cha, Su Jung Park, Hunseung Kang, Woe-Yeon Kim, Sang Yeol Lee, David Mackey, Min Gab Kim

**Affiliations:** 1 College of Pharmacy and Research Institute of Pharmaceutical Science, PMBBRC, Gyeongsang National University, Jinju, 660-701 Republic of Korea; 2 Division of Applied Life Sciences (BK21 Plus program), Graduate School of Gyeongsang National University, Jinju, 660-701 Republic of Korea; 3 Division of Applied Life Science (BK21Plus), PMBBRC & IALS, Gyeongsang National University, Jinju, 660-701 Korea; 4 Department of Plant Biotechnology, College of Agriculture and Life Sciences, Chonnam National University, Gwangju, 500-757 Korea; 5 Department of Molecular Genetics, Ohio State University, Columbus, Ohio 43210 USA; 6 Plant Breeding Institute, School of Life and Environmental Sciences, Faculty of Science, The University of Sydney, Narrabri, NSW 2390 Australia

## Abstract

In eukaryotes, RNA silencing, mediated by small interfering RNAs, is an evolutionarily widespread and versatile silencing mechanism that plays an important role in various biological processes. Increasing evidences suggest that various components of RNA silencing pathway are involved in plant defense machinery against microbial pathogens in *Arabidopsis thaliana*. Here, we show genetic and molecular evidence that Arabidopsis *SDE5* is required to generate an effective resistance against the biotrophic bacteria *Pseudomonas syringae* pv. *tomato* DC3000 and for susceptibility to the necrotrophic bacteria *Erwinia caratovora* pv. *caratovora*. *SDE5*, encodes a putative mRNA export factor that is indispensable for transgene silencing and the production of *trans*-acting siRNAs. *SDE5* expression is rapidly induced by exogenous application of phytohormone salicylic acid (SA), methyl jasmonate (MeJA), phytopathogenic bacteria, and flagellin. We further report that SDE5 is involved in basal plant defense and mRNA export. Our genetic data suggests that SDE5 and Nonexpressor of PR Gene1 (NPR1) may contribute to the same SA-signaling pathway. However, *SDE5* over-expressing transgenic plant exhibits reduced defense responsive phenotype after flagellin treatment. Taken together, these results support the conclusion that SDE5 contributes to plant innate immunity in *Arabidopsis*.

## Introduction

Plants have evolved potent inducible immune response to multiple pathogen attacks and bacterial pathogens provide a useful example of how pathogens are encountered at various levels. The first layer of defense responses is originated by perception of conserved molecular features of microbes, termed pathogen-associated molecular patterns (PAMPs)^1, 2^. PAMPs activate pattern-recognition receptors (PRRs), which in turn initiate diverse downstream signaling events that ultimately result in the activation of a basal resistance that is called PAMP-triggered immunity (PTI)^3, 4^. Bacterial molecules containing PAMPs include flagellin (the major protein of bacterial flagellum), lipopolysaccharides and the bacterial translation elongation factor, EF-Tu^1^. Flg22, a conserved 22 amino-acid peptide derived from the N terminus of *Pseudomonas syringae* flagellin^5^, is perceived by the receptor flagellin insensitivity 2 (FLS2) and subsequently activates downstream events such as mitogen-activated protein kinase (MAPK) cascades and WRKY transcription factors in Arabidopsis (*Arabidopsis thaliana*)^6, 7^. Bacteria counteract PTI by secreting defense-suppressing virulence effectors into host cells. As a counter defense strategy, host plants have evolved a repertoire of immune receptors, called disease resistance (R) proteins that can sense effectors and elicit effector-triggered immunity (ETI)^3, 4^. Both PTI and ETI are associated with the accumulation of defense signal molecules such as salicylic acid (SA), ethylene (ET), and jasmonic acid (JA). In Arabidopsis, SA-regulated defense responses including *Pathogenesis*-*Related* (*PR*) gene expression require the function of *Nonexpressor of PR Gene1* (*NPR1*) gene, which encodes a 66-kD protein with ankyrin repeats^8^.

RNA silencing is an RNA-guided, evolutionarily widespread, and versatile silencing mechanism that controls gene expression at the transcriptional (TGS, Transcriptional Gene Silencing) and post-transcriptional (PTGS, Post-transcriptional Gene Silencing) levels. In plants, RNA silencing is triggered by double-stranded RNA (dsRNA), processed into 21- to 24-nt short interfering (si)RNA or micro (mi)RNA by RNaseIII-like enzymes called Dicer-like proteins named DCL1–4^9, 10^. These small RNAs guide suppression of their target gene expression at the level of transcription, RNA stability or translation. RNA-induced silencing complexes invariably contain one member of the Argonaute (AGO) protein family^11–14^.

In plants, small RNAs including miRNAs and siRNAs regulate diverse processes including development, abiotic stress tolerance and defenses. Increasing studies indicate that host endogenous small RNAs and small RNA pathway components also participate in plant disease resistance against various pathogens, including bacteria, fungi, oomycetes and viruses. For example, in Arabidopsis, miR393 negatively regulates auxin signaling pathways and contributes to PTI^15^. Besides miR393, two other miRNA families, miR160 and miR167, are upregulated following *Pseudomonas syringae* pv. *tomato* (*Pto*) DC3000 infection and target members of auxin-response factors^16^. Although plants contain only several hundred miRNAs, they contain huge numbers of endogenous siRNAs, but only in a few cases the involvement of siRNAs in plant immunity has been described. In Arabidopsis, the natural antisense transcript (NAT)-derived endogenous nat-siRNAATGB2 and AtlsiRNA-1 are induced by the bacterial pathogen *Pto* DC3000 carrying an effector, *AvrRpt2*. These siRNAs play an important role in ETI by targeting negative regulators of the cognate *R* gene *RPS2* signaling pathway^17, 18^. Consistent with a role of these small RNAs in plant immunity, proteins required for small RNA biogenesis and function, such as DCL1, Hua Enhancer1 (HEN1) and AGOs family, have been shown to be required for disease resistance to bacterial pathogens^18–24^.

Previous studies indicated that SDE5, a homologue of a human mRNA export protein, is an essential component of the *trans*-acting siRNA pathway and is required for sense transgene PTGS (S-PTGS) but not inverted repeat transgene-mediated PTGS (IR-PTGS)^25, 26^. Mutation in *SDE5* also resulted in hyper-susceptibility to cucumber mosaic virus but not turnip mosaic virus^25^. However, the molecular mechanism by which SDE5 participates in plant defense system remains to be elucidated. Here, we report that SDE5 contributes to plant innate immunity in *Arabidopsis* via ETI pathway and suppresses PTI, while it could be induced by PAMP.

## Results

### *SDE5* gene expression is upregulated upon SA, MeJA and flg22 application and *Pto* DC3000 inoculation

**Figure 1:**
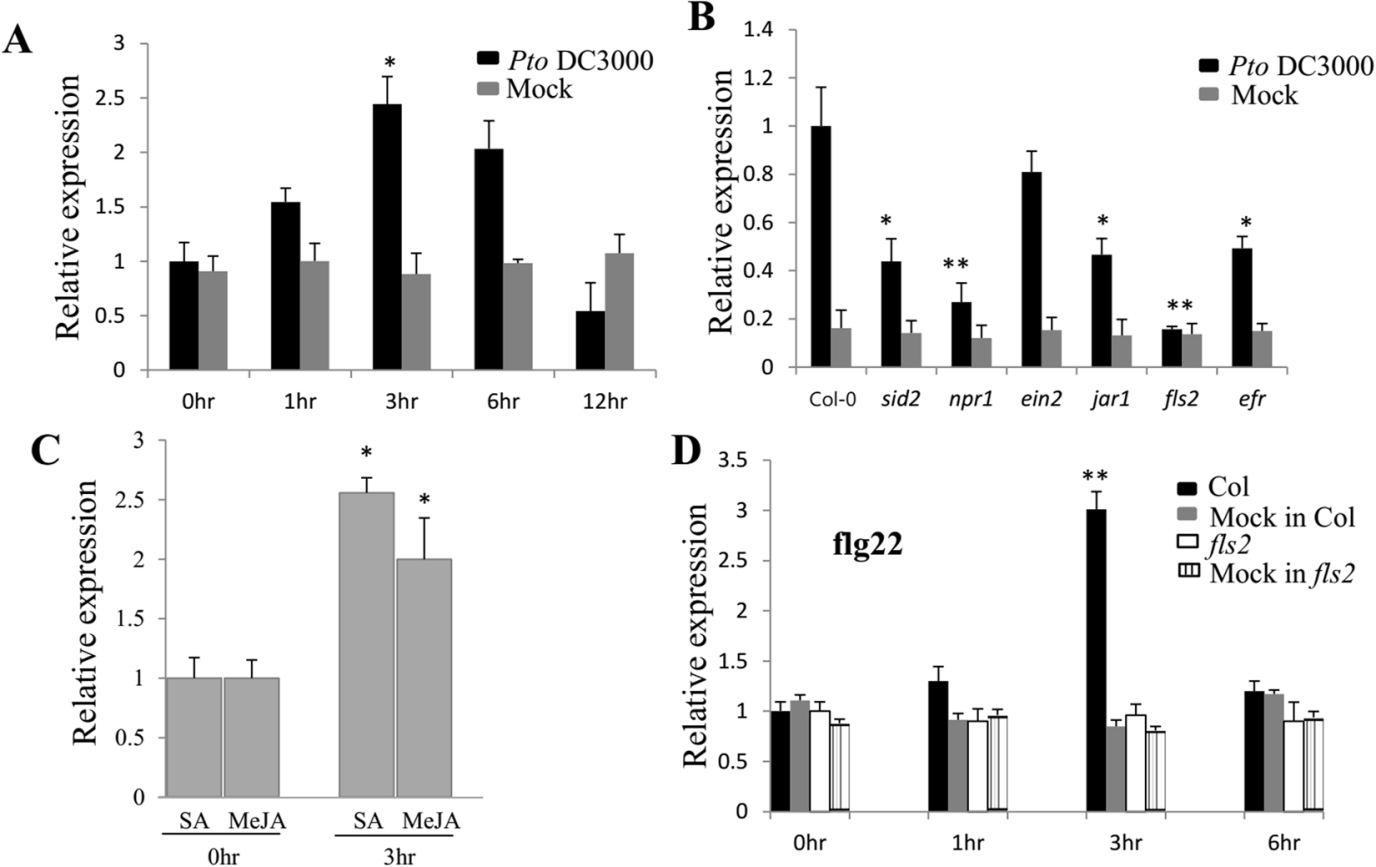
Expression analyses of the *SDE5* gene in response to pathogen inoculation, PAMP and hormonal treatments. (**A**) Fifteen-day-old Arabidopsis WT (Col-0) seedlings were vacuum infiltrated with 10 mM MgCl_2_ (Mock) or *Pto* strain DC3000 at 2 × 10^6^ cfu mL^−1^. Samples were collected 0, 1, 3, 6 and 12 h post infiltrated (hpi) and the transcript levels of the *SDE5* were determined by quantitative reverse-transcription polymerase chain reaction (qRT-PCR). (**B**) *SDE5* gene expression was monitored at 3 h upon *Pto DC3000* inoculation in mutants defective in SA production (*sid2*) or JA (*jar1*) and ethylene (*ein2*) signaling pathways, and also mutants altered in flagellin (*fls2*) or EF-Tu (*efr*) perception. (**C**) *SDE5* gene expression was examined at 3 h spraying with plant hormones, SA (250 μM), MeJA (200 μM) using fifteen-day-old WT seedlings. (**D**) Kinetics of *SDE5* gene expression in WT and the *fls2* mutant in response to exogenous application of flg22. Fifteen-day-old seedlings grown on MS medium were elicited using water (mock) or 10 μM of flg22 and harvested at 0, 1, 3 and 6 h. *ACTIN* was used as an internal control. Error bars indicate the mean ± SD for each set of three independent experiments with significant difference at *P < 0.05 and **P < 0.01.

To determine the expression of *SDE5* during plant basal defense, wild-type (WT) seedlings were infiltrated with the virulent *Pto* DC3000 at 2 × 10^6^ colony-forming units per mL (cfu mL^−1^) and *SDE5* transcript levels were then analyzed using quantitative reverse transcription-polymerase chain reaction (qRT-PCR) at different time points. As shown in Fig. 1A, *SDE5* expression was significantly up-regulated at 3 hours post inoculation (hpi). This upregulation was transient as gene expression returned to resting levels by 12 hpi.

To analyze the signaling pathway that leads to *SDE5* expression, we examined pathogen-induced changes of *SDE5* transcript levels in WT, as well as in mutants altered in the production of SA (*sid2*), JA signaling (*jar1*) and ethylene perception (*ein2*) following pathogen *Pto* DC3000 challenge. We also considered mutation in NPR1, one of the central downstream regulators of SA signaling. As shown in Fig. 1B, the *Pto* DC3000-induced expression of *SDE5* is unchanged in *ein2* suggesting that the induction of *SDE5* by the virulent bacterial pathogen is independent on EIN2. Interestingly, the level of *SDE5* expression was significantly reduced in *sid2*, *jar1* and *npr1*, indicating dependency on SA- and JA-signaling (Fig. 1B). The contribution of two PAMP receptors (FLS2 and EFR), involved in perception of flg22 and the elongation factor EF-Tu, respectively, to *SDE5* expression were evaluated using their respective mutants. The *Pto* DC3000-induced expression of *SDE5* was also reduced in the PAMP receptor-defective mutants (*fls2* and *efr*) indicating a contribution of PTI (Fig. 1B). To further validate the involvement of SA and JA in the induction of *SDE5*, we tested WT plants 3 h after spraying with SA and MeJA and observed that *SDE5* expression is 2–2.5-fold up-regulated (Fig. 1C).

The results obtained in Fig. 1B and previous reports^27, 28^, suggest a critical involvement of the FLS2-dependent signaling pathway in control of *SDE5* expression. To confirm the induction of SDE5 during PTI, we next monitored the mRNA levels of *SDE5* over a 6 h time course experiments. We found a significant induction of *SDE5* expression at 3 h after flg22 peptide treatment (Fig. 1D). As expected, expression was not induced by flg22 in the *fls2* mutant indicating that an important role of FLS2 in flg22 mediated *SDE5* induction. Taken together, *SDE5* expression data indicate a potential role for SDE5 in SA signaling, JA signaling and PTI in Arabidopsis.

### Disruption of *SDE5* decreases plant basal defense

**Figure 2:**
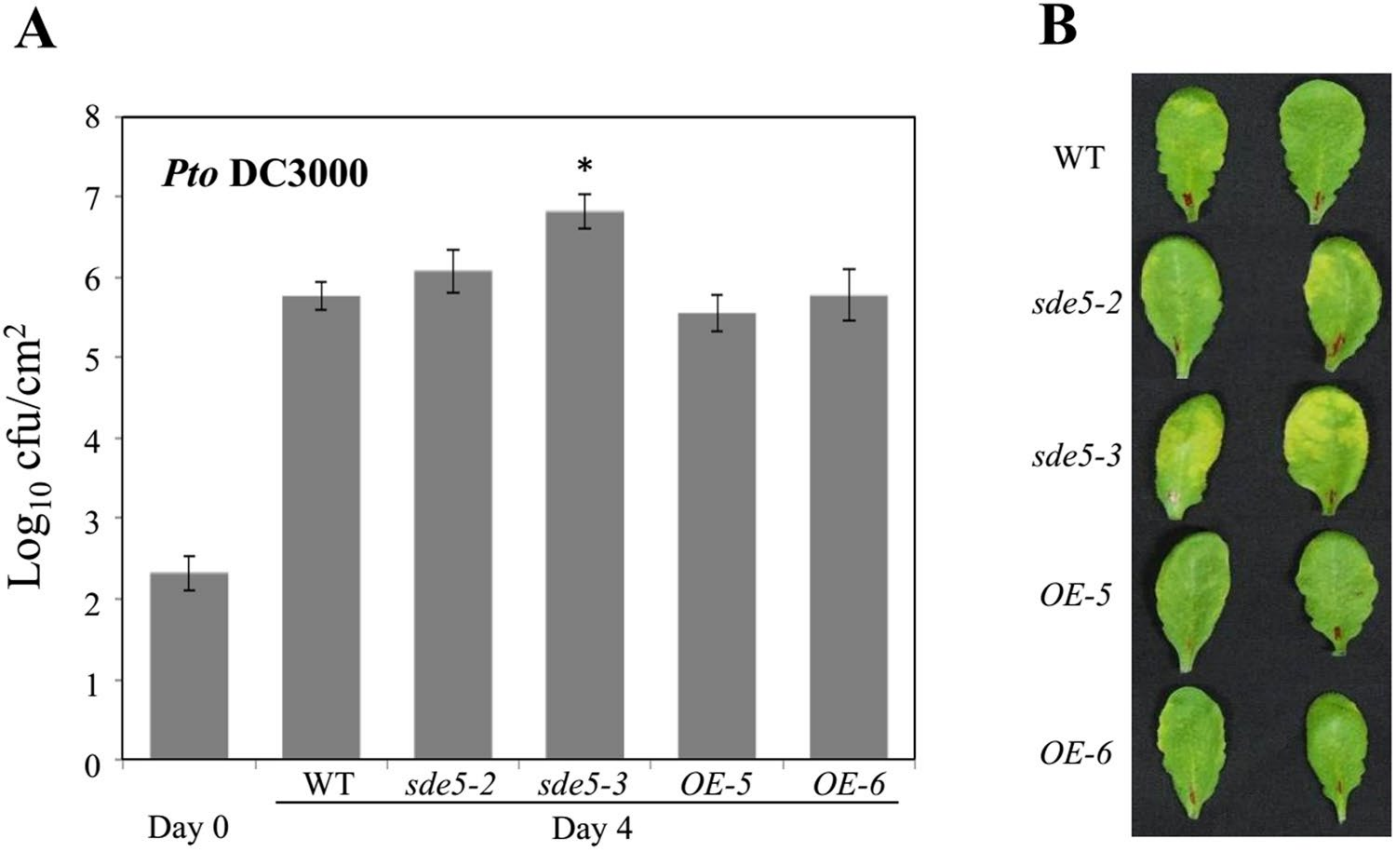
Altered susceptibility to *Pto* DC3000 in *sde5*-*3* mutant and transgenic line over-expressing the *SDE5* gene. (**A**) Quantification of bacterial growth 0 or 4 days post-infiltration (dpi) on five-week-old plants after syringe inoculation with concentrations of 2 × 10^5^ cfu mL^−1^ of the virulent bacterial strain *Pto* DC3000. The error bars indicate the mean ± SD for each set of three independent experiments with significant difference at *P < 0.05. (**B**) Disease symptoms in leaves of WT, *sde5*-*2*, *sde5*-*3* and *SDE5* overexpressing lines (*OE*-*5 and OE*-*6*) caused by *Pto* DC3000 infiltration. Leaves of five-week-old plants were syringe infiltrated with a concentration of 2 × 10^6^ cfu mL^−1^ of *Pto* DC3000, and photographs were taken 4 dpi. Representative leaves are shown. Similar results were obtained in three independent experiments.

In order to further elucidate the possible role of SDE5 in plant-pathogen interactions, a reverse genetic approach using mutant alleles of the *SDE5* gene containing a T-DNA insertion has been performed. The T-DNA insertion is in the sixth exon of the *SDE5* gene and resulted in the loss of detectable *SDE5* transcript, indicating that *sde5*-*3* ^26^ is a loss-of function mutant. Another mutant line used in this work was *sde5*-*2* which is known to be a partial loss-of-function mutant in mRNA export^25^. We also generated SDE5 overexpressing transgenic plants in the *sde5*-*3* background using *SDE5* coding sequence under the control of the *Cauliflower mosaic virus*-derived 35S promoter (Fig. S1). Two transgenic lines showing high expression of *SDE5* were selected for further study and named *OE*-*5* and *OE*-*6*. Then, we inoculated WT, *sde5*-*2*, *sde5*-*3* and two overexpressing lines, *OE*-*5* and *OE*-*6*, with a biotrophic pathogen, *Pto* DC3000, and monitored both bacterial growth (at 2 × 10^5^ cfu mL^−1^) and disease symptom development (at 2 × 10^6^ cfu mL^−1^). As shown in Fig. 2A, *sde5*-*3* permitted nearly 10-fold more bacterial growth than the WT plants. The *sde5*-*3* plants also developed significantly more severe disease symptoms than WT plants at 4 dpi (Fig. 2B). However, bacterial growth and disease development in partial loss-of-function mutant *sde5*-*2* were not significant compared with WT. As expected, this disease phenotype has been overcome in *OE* plants indicating that SDE5 may act as a positive regulator in plant innate immunity.

**Figure 3:**
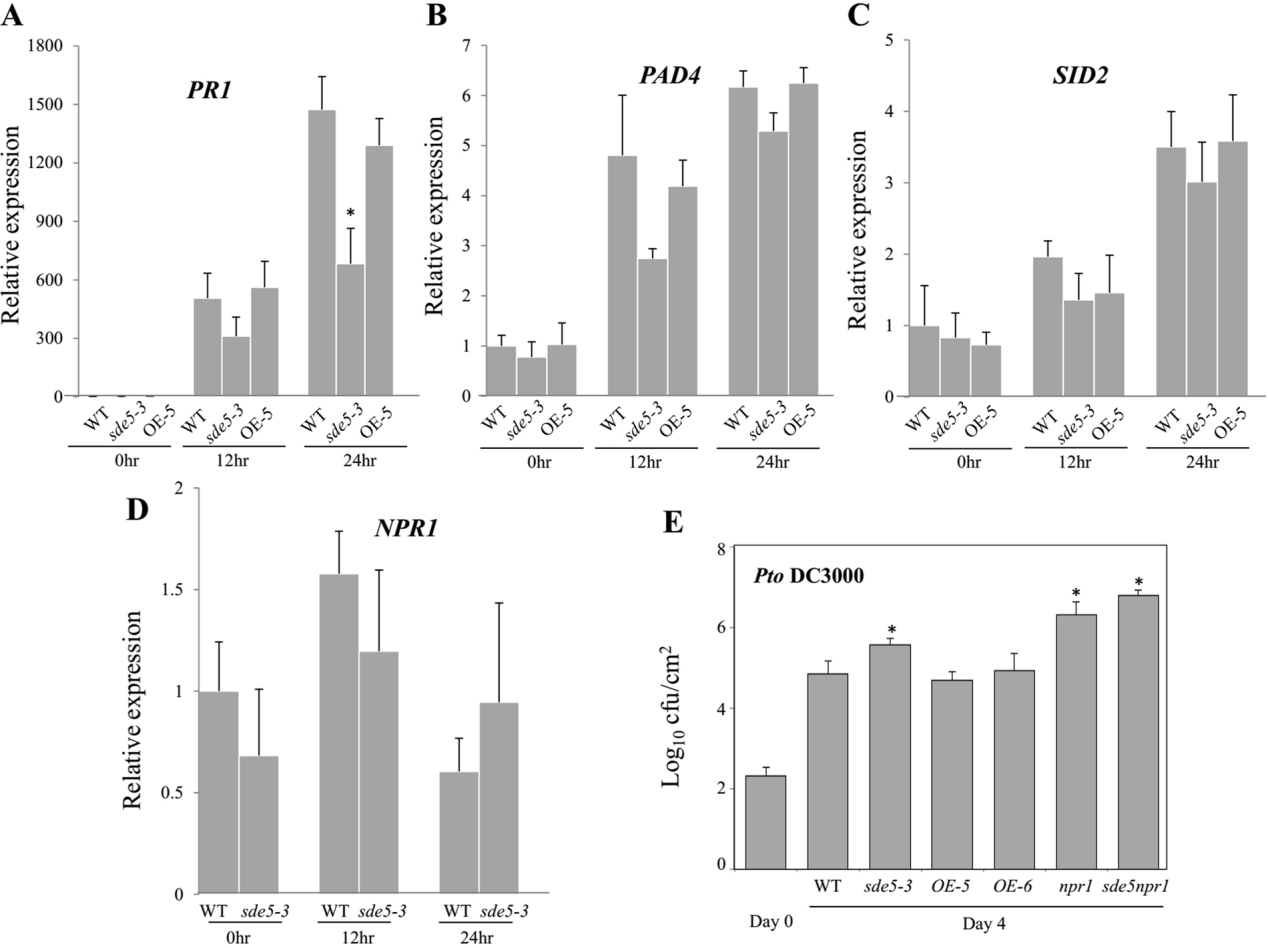
Expression analyses of defense marker gene and SA signaling pathway components upon *Pto* DC300 inoculation. (**A**) Analyses of *PR1* marker gene in various genetic backgrounds. Fifteen-day-old seedlings of WT, *sde5*-*2*, *sde5*-*3* and *SDE5* overexpressing lines (*OE*-*5 and OE*-*6*) were vacuum infiltrated with concentrations of 2 × 10^6^ cfu mL^−1^ of the *Pto* DC3000 and harvested at 0, 12 and 24 hpi. *ACTIN* was used as an internal control. (**B**–**D**) Expression of genes associated with SA mediated defense signaling pathway such as *PAD4* (**B**), *SID2* (**C**), and *NPR1* (**D**) using seedlings of plants in A. Error bars represent the standard error from three biological replicates. E, Bacterial growth assay of *Pto* DC3000 in WT, *sde5*-*3*, *OE*-*5*, *npr1*, and *sde5*-*3npr1* double mutant plants after syringe inoculation with concentrations 2 × 10^5^ cfu mL^−1^. The error bars indicate the mean ± SD for each set of three independent experiments with significant difference at *P < 0.05.

SA is a major plant defense hormone, central to the activation of a range of defenses including the induction of *PR* (pathogen-related) genes, systemic acquired resistance, and hypersensitive response^29^. Therefore, expression analysis of the well-known SA-dependent effector gene, *PR1*, was also performed in leaves of WT, *sde5*-*3* mutant and *OE*-*5* plants before and at various time points after inoculation with *Pto* DC3000. The result indicated that *PR1* transcript accumulation was significantly reduced in *sde5*-*3* compared with WT plants (Fig. 3A). Consistent with these findings, analysis of both loss-of-function *SDE5* mutants and gain-of-function *SDE5*-overexpressing plants indicates that SDE5 contributes to SA mediated plant basal defense by modulating responses to bacterial strain *Pto* DC3000. The biosynthesis of SA is strongly induced upon pathogen infection. This pathogen-induced SA biosynthesis is believed to be controlled by several key components including PAD4, EDS5 and SID2^30–32^. To further clarify the roles of SDE5 in SA-mediated basal defense, we examined the expression of *PAD4* and *SID2*, in WT, *sde5*-*3* mutant and *OE*-*5* plants following *Pto* DC3000 inoculation. No significant difference was found between WT plants and other genotypes tested (Fig. 3B and C), supporting the hypothesis that the effect of SDE5 on PR-1 expression is independent of expression of genes involved with SA production and signaling. To study the epistatic relationships between SDE5 and SA, we also treated SA before *Pto* DC3000 inoculation in Col-0, *sde5*-*2*, *sde5*-*3*, *OE*-*5* and *npr1*, and monitored symptoms. Disease symptom of *sde5*-*3* was not rescued by SA treatment suggesting that SDE5 is acting downstream of SA signaling rather than affecting SA biosynthesis (Fig. S2).

The SA-mediated signaling pathway regulated by NPR1 is one of the most important pathways in plant defense^33, 34^. The observed down-regulation of *SDE5* gene expression in *npr1* mutant plants indicates that functional NPR1 protein is required for pathogen responsiveness of *SDE5* (Fig. 1B). This result prompted us to investigate whether the accumulation of *NPR1* transcript levels are altered in WT and *sde5*-*3* mutant upon *Pto* DC3000 inoculation. However, no significant difference in the *NPR1* expression level was observed between the plants tested, indicating that SDE5 may function downstream of NPR1 in SA-signaling (Fig. 3D). Moreover, when challenged with virulent pathogens, the *sde5*-*3/npr1* double mutant was not more susceptible to *Pto* DC3000 than *npr1* (Fig. 3E), indicating that SDE5 and NPR1 might contribute to the same SA-signaling pathway in Arabidopsis.

### The *sde5*-*3* mutant confers elevated disease resistance to *ECC*

**Figure 4:**
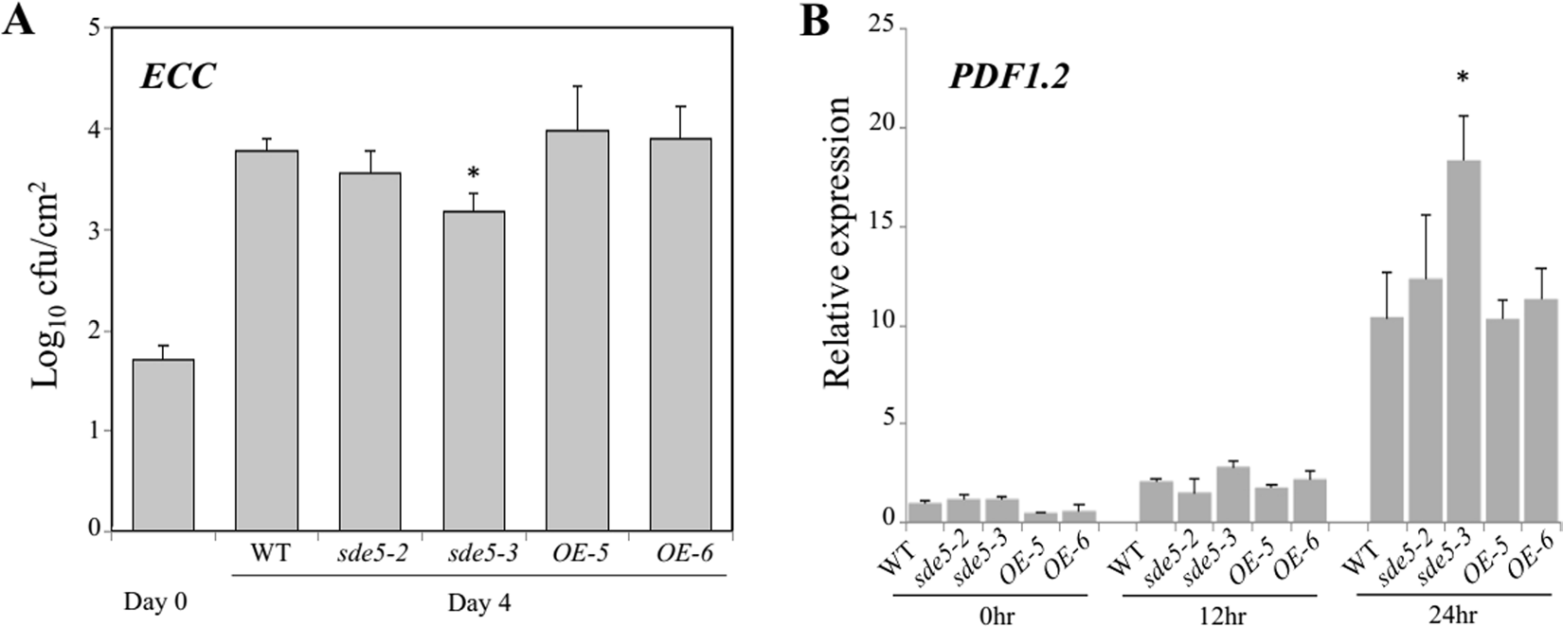
*sde5* potentiates the local disease response to *ECC* infection. (**A**) Bacterial proliferation assays. Five-week-old WT, *sde5*-*2*, *sde5*-*3* and *SDE5* overexpressing lines (*OE*-*5 and OE*-*6*) were inoculated with *ECC* at 2 × 10^5^ cfu mL^−1^. Leaf discs were collected after 7dpi and observed bacterial accumulation. The graph shows a representative result out of three independent experiments. (**B**) Quantitative analyses of *PDF1*.*2*, a gene associated with necrotrophic pathogen in plant defense. Fifteen-day-old seedlings stated in A vacuum infiltrated with a concentration of 2 × 10^6^ cfu mL^−1^ of *ECC* were harvested at 0, 12 and 24 hpi. *ACTIN* is used as internal control. The error bars indicate the mean ± SD for each set of three independent experiments with significant difference at *P < 0.05.

Since, *SDE5* expression was induced by exogeneous MeJA treatment, we evaluated the contribution of SDE5 to plant responses to the necrotrophic pathogen, *Erwinia caratovora* pv. *caratovora* (*ECC*). We inoculated WT, *sde5*-*2*, *sde5*-*3*, *OE*-*5* and *OE*-*6* plants with *ECC* and observed bacterial accumulation at 4 dpi. As shown in Fig. 4A, *sde5*-*3* mutant plants restricted the growth of *ECC* relative to WT plants, indicating that *SDE5* promotes the growth of *ECC*. *ECC* susceptibility was restored to WT levels in the *OE*-*5* and *OE*-*6* plants, further confirming that expression of the *SDE5* gene promotes infection by this necrotrophic bacterial (Fig. 4A). In an effort to further examine the role of *SDE5* in JA-mediated defenses, we determined the expression levels of plant defensin gene *PDF1*.*2*, a characteristic molecular response of plants to necrotrophic pathogen attack in different time points after pathogen inoculation. The induction of *PDF1*.*2* was significantly increased in *sde5*-*3* plants following the inoculation with *ECC* (Fig. 4B). This observation is congruent with the observation that *sde5*-*3* plants showed enhanced disease resistance to this pathogen. Collectively, these results indicate that SDE5 plays positive and negative roles in SA- and JA-mediated pathogen defense, respectively, possibly by participating in the cross-talk between these signaling pathways.

### SDE5 is involved in PAMP triggered immunity in Arabidopsis

**Figure 5:**
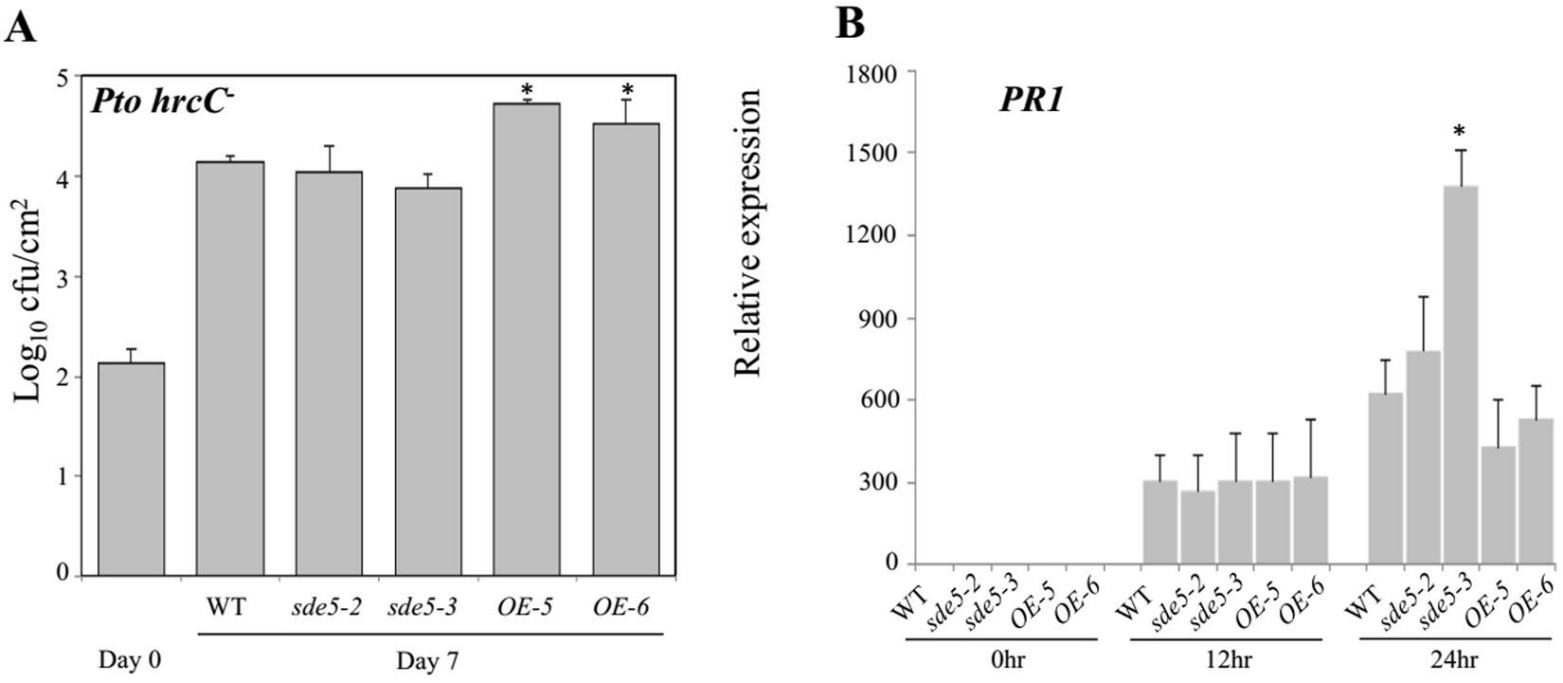
Altered responses were displayed by the *sde5* mutants and the transgenic lines over-expressing the *SDE5* upon TTSS-defective mutant *Pto hrcC* ^*−*^ strain. (**A**) Quantifications of *in planta* bacterial growth in the Arabidopsis genotypes as indicated were performed at 0 or 7 dpi using at 2 × 10^5^ cfu mL^−1^ of *hrcC* ^*−*^. Plants were placed under high humidity condition (in dew chamber) after infiltration for this experiment. The error bars indicate the mean ± SD for each set of three independent experiments with significant difference at *P < 0.05. Data are representative of four replicates of three independent experiments. (**B**) Quantitative analyses of *PR1* using fifteen-day-old seedlings stated in (**A**) vacuum infiltrated with a concentration of 2 × 10^6^ cfu mL^−1^ of *Pto hrcC* ^*−*^. Samples were harvested at 0, 12 and 24 hpi. Each bar represents the relative expression of the genes compared with the *ACTIN* control. Similar results were obtained in independent experiments. Asterisk (*) indicate significant difference at a *P* value < 0.05.

SA is required for the full activation of PTI^35, 36^. To better understand the contribution of SDE5 in PTI, we utilized the type-three secretion system (TTSS)-defective mutant *Pto* DC3000 *hrcC* ^*−*^ (*Pto hrcC* ^*−*^) strain, which can elicit, but not suppress, PTI responses due to its inability to inject effector proteins within host cells^37^. The behavior of WT, *sde5*-*2*, *sde5*-*3*, *OE*-*5* and *OE*-*6* plants was analyzed following foliar inoculation with the *Pto hrcC* ^*−*^ strain, and, as expected, limited bacterial growth was observed in WT plants compared to the fully virulent bacteria following inoculation at 2 × 10^5^ cfu mL^−1^. No significant differences were detected between WT and the *sde5* mutant lines (Fig. 5A). In contrast, *OE*-*5* and *OE*-*6* plants were more susceptible to *Pto hrcC* ^*−*^ relative to the WT (Fig. 5A). We next examined the expression pattern of *PR1* in response to *Pto hrcC* ^*−*^ inoculation in various genotype plants. As shown in Fig. 5B, *PR1* gene expression was highly induced in *sde5*-*3* plants with significant up-regulation at 24 hpi in comparison to other plants. These results reveal an opposite regulation of *PR1* gene expression by *SDE5* following infection with virulent *Pto* DC3000 versus the TTSS-deficient *Pto hrcC* ^*−*^ strain. These data further support the hypothesis that the responses observed with *Pto* DC3000 are TTSS-dependent and therefore may involve the activity of bacterial effectors in plant cells. Thus, SDE5 restricts susceptibility to virulent *Pto* DC3000, but has a less discernible effect on resistance to *Pto hrcC* ^*−*^ that cannot deliver effectors.

**Figure 6:**
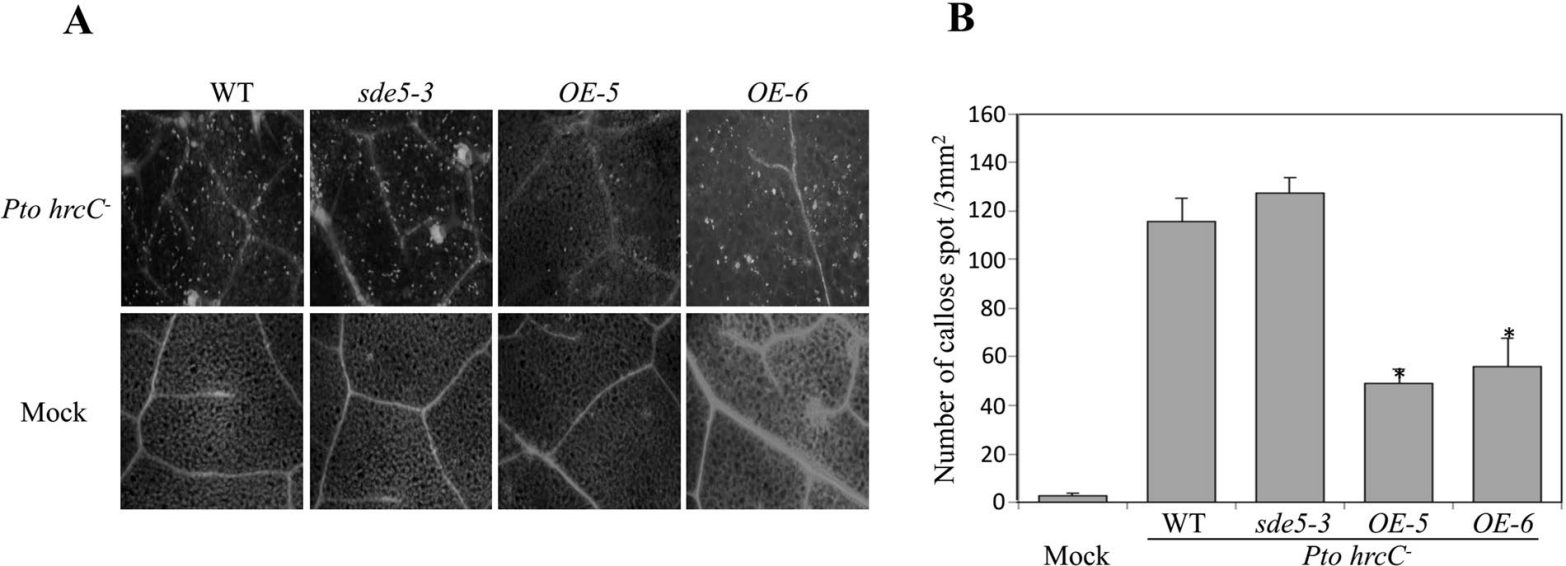
Callose deposition significantly reduced in transgenic overexpressing plants upon *hrcC* ^*−*^ infection. (**A**) Callose detection was observed using leaves of four-week-old WT, *sde5*-*3* and *OE*-*5* plants infiltrated with *Pto hrcC* ^*−*^ bacteria (upper panel) and 10 mM MgCl_2_ (mock; lower panel). Staining was performed at 16 hpi. (**B**) Quantitative analyses of callose deposition on plants used in A using aniline blue staining. Experiments were performed using four leaves harvested from different plants for each genotype. The error bars indicate the mean ± SD for each set of three independent experiments with significant difference at *P < 0.01.

The observed results indicate that SDE5 may negatively regulate PTI. We further analyzed the formation of cell wall depositions of callose, a PTI response that plays a critical role in the establishment of basal immunity^38–40^ in different genotype upon *Pto hrcC* ^*−*^ inoculation. Callose accumulated significantly less in *OE*-*5* and *OE*-*6* plants compared with WT and *sde5*-*3* mutant plants, reinforcing a negative role for SDE5 in PTI response (Fig. 6).

Next, a model bean (*Phaseolus vulgaris*) pathogen *P*. *syringae* pv. *phaseolicola* 1448a^41^, *Pph*, that is unable to efficiently suppress defense reactions in Arabidopsis was further introduced^42, 43^. Forsyth *et al*.^44^ established that a major determinant of non-host resistance to *Pph* in Arabidopsis is FLS2. Therefore, the pathosystem *Arabidopsis–Pph* is considered a classical model to study FLS2-mediated defenses and PTI. We examined the behavior of *sde5*-*3* genotypes upon inoculation with the *Pph* strain. Significantly enhanced bacterial growth was observed in the transgenic *OE*-*5* plant compared to WT (Fig. S3) at 7 dpi. This is consistent with the susceptibility of *OE*-*5* and *OE*-*6* plants to *Pto hrcC* ^*−*^ strain and confirms that SDE5 restricts PTI-dependent defense system.

### Transgenic overexpressing plant exhibits altered molecular and cellular responses to flagellin application

**Figure 7:**
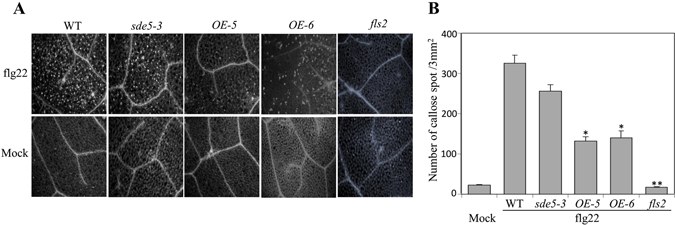
Callose deposition is significantly reduced in transgenic overexpressing plants upon flg22 treatment. (**A**) Detection of callose papillae on leaves of WT, *sde5*-*3*, *OE*-*5* and the *fls2* mutant plants infiltrated with water (mock; lower panel. and 10 μM flg22 (upper panel) at 16 hpi. (**B**) Number of callose papillae was quantified on plants used in A using aniline blue staining. Four leaves harvested from different plants for each genotype. The error bars indicate the mean ± SD for each set of three independent experiments with significant difference at *P < 0.05 and **P < 0.01.

The up-regulation of *SDE5* transcript by exogenous flg22 treatment prompted us to examine the function of SDE5 protein in the FLS2 signaling pathway. Because callose formation is induced in response to PAMPs^45^, phenotypic assays for flg22-induced callose deposition were performed on *sde5*-*3* genotypes^46, 47^. Callose accumulation is undetectable in *fls2*. And, as was observed with *Pto hrcC* ^*−*^ inoculation, flg22 induced callose accumulation is significantly reduced in transgenic *OE*-*5* and *OE*-*6* plants compared to WT and *sde*-*5* plants (Fig. 7). These results suggest that SDE5 has a negative role in the production and/or deposition of callose probably by regulating FLS2-mediated signaling pathway.

The perception of flagellin by plant cells also leads to important changes in gene expression^27, 28^. Therefore, we next analyzed the transcript level of two PTI marker genes including *Flg22 RECEPTOR KINASE 1* (*FRK1*) and *WRKY29*, using mRNA from seedlings of WT, *sde5*-*3*, *OE*-*5* and *OE*-*6* lines prepared at 3 h before and after 1 μM flg22 treatment. Both *FRK1* and *WRKY29* transcripts increased in WT seedlings, but not in *fls2* mutant, after flg22 elicitation, confirming that these genes are PAMP-responsive. Transcript accumulation of *FRK1* and *WRKY29* was similar among WT, *sde5*-*3*, *OE*-*5* and *OE*-*6* lines after flg22 treatment (from 0 to 3 h) (Fig. S4). These results indicate that SDE5 involves in the FLS2-signaling pathway at downstream or independently of the induction of these PAMP-responsive genes.

### SDE5 is required for mRNA export

**Figure 8:**
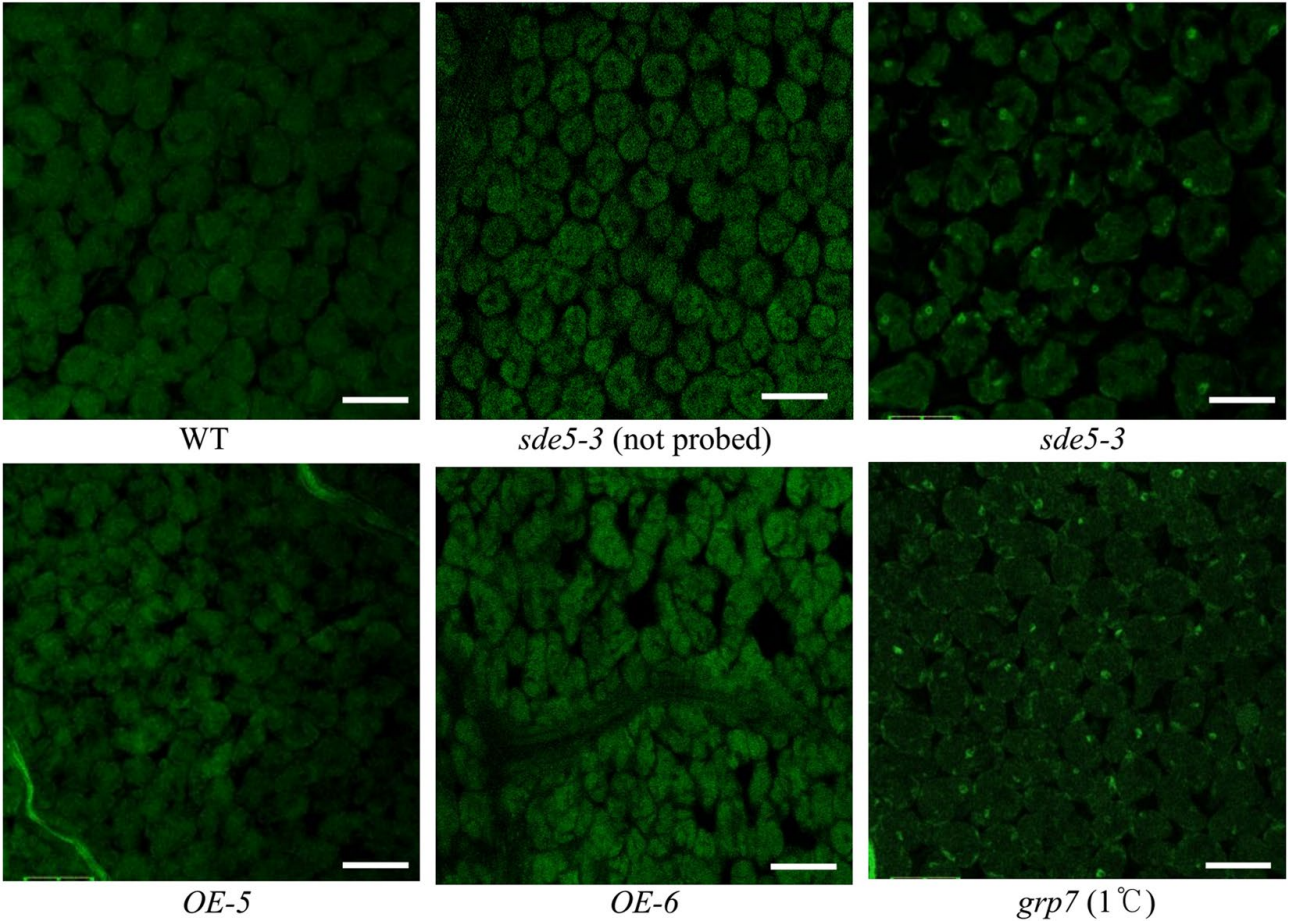
mRNA export is impaired in *sde5*-*3* plants. Small leaf discs from fifteen days old WT, *sde5*-*3*, *OE*-*5* and *grp7* (cold treated for 2 days) plants were fixed and probed with a fluorescently labeled oligo(dT) probe. The samples were observed under an OLYMPUS 1 × 71 FV500 confocal laser-scanning microscope Green spots represent accumulation of mRNA. In *sde5*-*3* and *grp7* cells, mRNA accumulates at much higher level in the nuclei. Each experiment was repeated at least three times, and similar results were obtained. Scale bar = 100 μm.

A previous report suggested that SDE5 has some similarity (12% identity and 58% similarity) to the C-terminal domain (PF03943) of human mRNA export factor TAP (or NXF1)^48^. The TAP C-terminal domain is particularly important for the function of TAP as an mRNA export mediator because it binds to nucleoporin complexes^48, 49^. Therefore, we speculated that mRNA export may also be affected in the *sde5*-*3* mutant. To test this hypothesis, we performed an *in situ* hybridization assay^50^ to localize poly(A) signals in WT and *sde5*-*3* mutant plants. The poly(A) signals were examined by confocal microscopy, using *grp7* as a positive control^51^. As shown in Fig. 8, the fluorescein poly(A) signals were stronger in the nuclei of *sde5*-*3* and *grp7* than WT, *OE*-*5* and *OE*-*6*, indicating that mRNA export is diminished in *sde5*-*3* plants, resulting in mRNA accumulation in the nucleus. The signal was not observed in not probed *sde5*-*3* mutant. This result indicates that Arabidopsis SDE5 is likely a contributing factor in the mRNA export pathway.

## Discussion

Our results convey that SDE5, a putative RNA export protein and an essential component of the *trans*-acting small interference RNA (tasiRNA) pathway, may have dual roles in plant defense mechanism. For instance, SDE5 acts as a positive regulator of plant defense system upon fully virulent *Pto* DC3000 strain (Figs 2 and 3). In contrast, less callose deposition and *PR1* expression in *SDE5*-overexpressing lines upon flg22 application or *Pto hrcC* ^*−*^ inoculation indicate that SDE5 may act as a negative regulator of the flagellin signaling pathway (Figs 6 and 7).

Our results show that the *SDE5* expression is rapidly induced in response to phytopathogenic bacteria and PAMP treatment (flagellin). Using a reverse genetics approach, we clearly demonstrated that SDE5 acts as a positive regulator of plant defense in response to biotrophic pathogen *Pto* DC3000. The knockout mutant exhibits higher levels of susceptibility than WT to *Pto* inoculations, and, conversely, SDE5 over-expressing transgenic lines exhibit fewer discernible symptoms and a significantly lower level of bacteria development compared to WT and knockout plants (Fig. 2). In addition, SA-regulated defense marker gene, *PR1* expression was significantly downregulated in *sde5*-*3* relative to WT and *OE*-*5* plants (Fig. 3A).

Signaling cross-talk between plant hormones, such as SA, ET and JA, fine tunes the plant defense response^52^. In general, it is believed that SA signaling plays an important role in resistance to biotrophic pathogens and ET/JA signaling plays a crucial role in resistance to necrotrophic pathogens^53^. And both synergistic and antagonistic interactions between SA and ET/JA signaling pathways have been reported^54^. Up-regulation of JA-mediated responses and inhibition of SA-inducible defenses result in enhanced resistance to necotrophs but increased susceptibility to biotrophs^55^. Data reported in this study also indicates that SDE5 regulates disease susceptibility to the necrotrophic pathogen, *ECC* and the expression of *PDF1*.*2* in an opposite way to the biotrophic pathogen (Fig. 4). Depending on the type of invader, a particular subset of defense responses might be activated, such as SA- and JA-mediated signaling pathways, to specifically fend off specific classes of pathogens.

To examine the role of SDE5 in PTI-mediated restriction of bacterial growth, we challenged Arabidopsis with two pathogens, *Pto hrcC* ^*−*^ and *Pph*. The *Pto hrcC* ^*−*^ bacteria trigger PTI and lack effectors to suppress it. FLS2-dependent PTI makes a critical contribution to the resistance of Arabidopsis to *Pph*, which lacks effectors required to efficiently block PTI^43, 44^. When either *Pto hrcC* ^*−*^ or *Pph* were introduced into WT, *sde5*-3 and over-expressing lines, the *OE*-*5* and *OE*-*6* plants showed increased susceptibility compared to WT control and *sde5*-*3* plants (Figs 5 and S3). These results align with the observation of reduced cell wall depositions of callose and lower *PR1* gene expression in the *OE*-*5* and *OE*-*6* plants (Figs 5 and 6). On the other hand, *sde5*-*3* showed an opposite tendency, as indicated by the increased *PR1* expression. Thus, SDE5 may have a negative role in the PAMP-signaling pathway resulting in reduced plant defense responses. Although SDE5 negatively regulates callose deposition, it did not affect the expression of early induced PTI marker genes such as *FRK1* and *WRKY29*, indicating that SDE5 might regulate a later stage of plant defense signaling (Fig. S4). In addition, the results presented here convey the involvement of SDE5 in PTI through a flagellin-dependent signaling pathway. Further research would be required in order to explore the molecular mechanism controlled by SDE5 in plant defense signaling.

Previous studies revealed that siRNA-related components, such as AGO4, DRB4 and HPR1, involve in plant defense system. A mutation in the *AGO4*, that is associated with siRNAs showed enhanced susceptibility to the bacterial pathogen *Pto* DC3000^19^. An important observation presented by the Lopez *et al*.^56^ showed that the co-existence of an enhanced disease resistance to a biotrophic bacteria, like *Pto* DC3000, with an enhanced susceptibility to necrotrophic fungi in RNA Polymerase V (Pol V)-defective mutant. Pol V is crucial for the RNA-directed DNA methylation (RdDM) pathway that is an epigenetic control mechanism driven by siRNAs. A mutation in double-stranded RNA binding protein 4 (DRB4) had a more severe effect on RPS2- and RPM1-mediated resistance to *Pto* DC3000 expressing *avrRpt2* or *avrRpm1*, respectively^57^. Biochemical studies also suggest that DRB4 is required for the stability of RPS2 and RPM1 proteins and thereby resistance mediated by these R proteins^57^. Similarly, an mRNA export factor in Arabidopsis, HPR1, which is also involved in the production of endogenous and exogenous siRNA, acts as a positive regulator in plant defense signaling^23^. Based on these findings, we hypothesize that SDE5 functions in plant defense system in the same pathway as other siRNA components above upon biotrophic pathogen infection.

Many studies also have shown that the mutants impaired in mRNA export exhibit enhanced susceptibility to pathogens^58^. Such as, in Arabidopsis, mutations in *MOS3* (*modifier of snc1*) and *MOS11* (*suppressor of npr1*-*1*, *constitutive 1*) lead to defects in mRNA export^58, 59^. Similar to *sde5*-*3* plants, both *mos3* and *mos11* single mutants are more susceptible to the virulent strain than WT^58, 59^. Pan *et al*.^23^ reported that HPR1, another mRNA trafficking protein, contributes to the basal defense against virulent pathogens. And accordingly, similar to *hpr1*, *mos3* and *mos11*, mRNA export was affected in the *sde5*-*3* mutant (Fig. 8). Consistent with these observations, we further conclude that SDE5, HPR1, MOS3 and MOS11 probably belong to the same pathway. Interestingly, *prl1* mutant, a loss-of-function mutant of a second SDE5 homologue in Arabidopsis (at AT58720)^60^, exhibited enhanced susceptibility to various kinds of virulent and avirulent pathogens^61^. Thus, it would be speculated that SDE5 and PRL1 may function together to regulate innate immunity in Arabidopsis.

In this study, we found that SDE5, a homologue of a human mRNA export protein, is upregulated upon pathogen inoculation, exogenous PAMP and hormonal application and *sde5* mutant plants are defective in mRNA export from the nucleus to cytoplasm. Loss of SDE5 function leads to increased susceptibility to the biotrophic pathogen *Pseudomonas syringae* and enhanced resistance toward necrotrophic *Erwinia caratovora* pv *caratovora* (*ECC*). In addition, knockout mutants and over-expressing transgenic plants also exhibit delayed defense responses after flg22 treatment. Taken together, our results establish that SDE5 contributes to plant innate immunity.

Although, we showed that SDE5 is involved in plant disease resistance, knowledge regarding the role of the mRNA export in plant defense responses is just emerging. Therefore, further functional analysis will be required to determine the detail molecular basis of mRNA export and defense responses in plants.

## Methods

### Plant lines, growth conditions and chemical treatments

Arabidopsis plants used in all experiments were derived from ecotype Columbia-0 (Col-0). Mutant lines were *sde5*-*2* ^25^, *sde5*-*3* ^26^, *sid2* ^32^, *ein2* ^62^, *jar1* ^63^ and *npr1* ^8^, and *fls2* obtained from the Arabidopsis Biological Resource Center. Genotyping of the Transfer DNA (T-DNA) insertion lines was performed by PCR, using allele-specific primers. The double mutant was produced by crossing the above mutants and genotyped using the primer sets listed in Supplemental Table S1. Plants were grown either on soil or on plates containing Murashige and Skoog (MS) medium (Sigma-Aldrich) with 1% sucrose and 0.6% agar (Sigma-Aldrich) in a growth chamber (16 h of dark and 8 h of light) unless otherwise indicated. SA (250 μM), MeJA (200 μM), flg22 (10 μM) and sterile water (for mock) treatment was carried out in fifteen-day-old seedlings grown in MS medium with 1% sucrose. Seedlings were collected as indicated time after treatment, immediately frozen in liquid nitrogen, and stored at −80 °C until RNA purification.

### Bacterial strains, growth conditions and inoculations

The *P*. *syringae* strains and *ECC* strain SCC1^64^ used in this study were grown at 28 °C on King’s B (KB) medium supplemented with the appropriate antibiotics: 50 μg/ml rifampicin and 50 μg/ml kanamycin (for *Pto* DC3000) or 50 μg/ml rifampicin (for *Pto hrcC* ^*−*^, *Pph* and *ECC*). Inoculation was performed as described^65^. In brief, five-week-old leaves were infiltrated with a needless syringe on the abaxial side at the indicated densities. Mock-treated plants were infiltrated with 10 mM MgCl_2_ alone. Disease symptoms and quantification of bacterial growth was performed at the indicated times. These experiments were repeated at least three times with similar results.

### Plasmid constructs and plant transformation

Constructs overexpressing *SDE5* were generated using the Gateway cloning system (Invitrogen). The *SDE5* coding sequence was amplified by PCR using cDNA synthesized from total RNAs of Arabidopsis WT Col-0 seedlings as the template. The amplified fragment was cloned into the pENTR/D-TOPO vector and inserts were confirmed by sequencing. The entry clones were subsequently transformed into the destination vector pSITE-4CA (to overexpress the protein fused to RFP). These constructs were transformed into WT plants through *Agrobacterium tumefaciens* (GV3101 strain)-mediated floral dip method^66^. Homozygous transgenic lines were selected and transgene expression was analyzed by qRT-PCR and by confocal microscopy to the *35* *S*::*SDE5*-*RFP* transgenic plants.

### Callose staining

Callose detection was performed as described^65^. In brief, four-week-old leaves were syringe-infiltrated with 2 × 10^6^ cfu/ml of *Pto hrcC* ^*−*^, 10 μM flg22, 10 mM MgCl_2_ (Mock for bacterium) and sterile water (Mock for flg22) and collected after 16 h. Whole leaves were collected, stained with 0.1% methyl blue, mounted in 50% glycerol, and examined with OPTIKA fluorescence microscope. Four leaves were prepared for each treatment. Three independent biological assays were performed. Representative views of these pictures were randomized, and the number of callose deposits was counted blind.

### Gene expression analyses

Total cDNA was synthesized from total RNA of fifteen-day-old seedlings either pathogen inoculation or chemical treatment as indicated using the SuperScript III first strand synthesis system (Invitrogen), according to the manufacturer’s instructions. qRT-PCR was performed using a Bio-Rad CFX96 Real-Time System. Amplification curves and gene expression were normalized using *ACTIN* as an internal standard. The primers used for qRT-PCR were listed in Supplemental Table S1. Triplicate biological and technical replications were performed. Data were analyzed using BioRad CFX Manager 2.0 Software.

### Poly(A) mRNA *in situ* localization assay

Poly(A) mRNA *in situ* hybridization was conducted essentially as described previously^50, 51^. Briefly, leaf samples of 2-week-old seedlings were fixed in a fixation buffer (3 mM NaH_2_PO_4_, 7 mM Na_2_HPO_4_, 120 mM NaCl, 2.7 mM KCl, 80 mM EGTA, 0.1% Tween 20, 5% formaldehyde, 10% DMSO, and 50% heptane), were subsequently incubated in 1:1 ethanol:xylene, and were washed with ethanol, methanol and finally with 1:1 methanol:fixation buffer. The samples were post-fixed in the fixation buffer for 30 min at room temperature, and were rinsed with Hyb Plus hybridization buffer (Sigma-Aldrich). After prehybridization in hybridization buffer for 1 h at 50 °C, 10 pmol of 45-mer oligo(dT) labeled with fluoresceine at the 5′-end was added and hybridized at 50 °C in darkness. The samples were then washed in 2 × SSC (1 × SSC is 0.15 M NaCl and 0.015 M sodium citrate), 0.1% SDS at 50 °C and in 0.2 × SSC, 0.1% SDS at 50 °C in darkness. The samples were immediately observed under an OLYMPUS 1 × 71 FV500 confocal laser-scanning microscope (Olympus America Inc.) with a 488-nm excitation laser and a 522/DF35 emission filter at identical laser strength. Each experiment was repeated at least three times, and similar results were obtained.

## Electronic supplementary material

Supplementary Information
