# Supplementary material for: SDE5, a putative RNA export protein, participates in plant innate immunity through a flagellin-dependent signaling pathway in Arabidopsis
Source: Sci Rep. 2017 Aug 29;7:9859. doi: 10.1038/s41598-017-07918-x (PMC5574965; doi:10.1038/s41598-017-07918-x)
Supplement: Supplementary file 1 — Supplementary Information [file 41598_2017_7918_MOESM1_ESM.docx]

**Supplemental Information**

**SDE5, a putative RNA export protein, participates in plant innate immunity through a flagellin-dependent signaling pathway in *Arabidopsis***

Mohammad Nazim Uddin**^1^** , Salina Akhter**^2,^**, Rupak Chakraborty**^1^**, Ji Hyeong Baek**^1^**, Joon-Yung Cha**^3^**, Su Jung Park**^4^**, Hunseung Kang**^4^**, Woe-Yeon Kim**^3^**, Sang Yeol Lee**^2^**, David Mackey**^5,*^**, Min Gab Kim**^1,*^**

^1^College of Pharmacy and Research Institute of Pharmaceutical Science, PMBBRC, Gyeongsang National University, Jinju 660-701, Republic of Korea.

**^2^**Division of Applied Life Sciences (BK21 Plus program), Graduate School of Gyeongsang National University, Jinju 660-701, Republic of Korea.

**^3^**Division of Applied Life Science (BK21Plus), PMBBRC & IALS, Gyeongsang National University, Jinju 660-701, Korea

**^4^**Department of Plant Biotechnology, College of Agriculture and Life Sciences, Chonnam National University, Gwangju 500-757 Korea

^5^ Department of Molecular Genetics, Ohio State University, Columbus, Ohio 43210. USA.

**Correspondence Author**

Min Gab Kim

E-mail : [mgk1284@gnu.ac.kr](mailto:mgk1284@gnu.ac.kr)

**Or**

David Mackey

E-mail : [mackey.86@osu.edu](mailto:mackey.86@osu.edu)


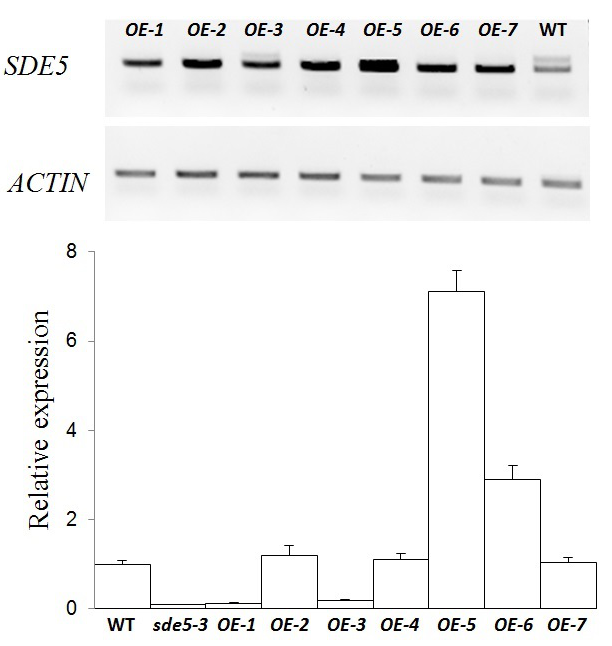


**Supplemental Figure 1.** Identification of SDE5 overexpressing lines. RT-PCR (upper panel) and qRT-PCR (lower panel) analysis of the *SDE5* expression in the WT, *sde5-3* and several *35S::SDE5-RFP* transgenic plants. *ACTIN* served as an internal control.

**Supplemental Figure 2.** *Pto* DC3000 induced symptoms after SA treatment in *sde5* mutants and transgenic lines over-expressing the *SDE5* gene. Symptoms in leaves of WT, *sde5-2, sde5-3* and *SDE5* overexpressing line (*OE-5*) treated with 0.5 mM SA followed by *Pto* DC3000 infiltration. Leaves of five-week-old plants were syringe infiltrated with a concentration of 2x10^6^ cfu mL^-1^ of *Pto* DC3000, and photographs were taken 4 dpi. Representative leaves are shown. Similar results were obtained in three independent experiments.

**Supplemental Figure 3.** Enhanced bacterial growth is observed in transgenic overexpressing plants upon non-host pathogen, *Pph* infection. Quantification of bacterial growth 0 or 7 dpi on five-week-old plants after syringe inoculation with concentrations of 2x10^5^ cfu mL^-1^ of the virulent bacterial strain *Pph*. The bars indicate the mean ±SD for each set of three independent experiments with significant difference at **P* < 0.05.

**Supplemental Figure 4.** Expression analysis of flg22-associated marker genes. A to B, qRT-PCR analyses of *FRK1* (A) and *WRKY29* (B) of seedlings WT, *sde5-3,* *OE-5* and the *fls2* pretreated for 3 h with 1 μM flg22 treatment. *ACTIN* was used as loading control. Values below each section represent relative abundance of transcript normalized to *ACTIN* control.The bars indicate the mean ±SD for each set of three independent experiments with significant difference at **P* < 0.01.
